# Latent myofascial trigger points injection therapy for adult cough variant asthma: A randomized controlled trial

**DOI:** 10.3389/fmed.2023.937377

**Published:** 2023-02-23

**Authors:** Qianqian Liu, Wenwen Zhang, Tian Tian, Yu Liu, He Bai, Qiya Hu, Feng Qi

**Affiliations:** ^1^Department of Anesthesiology and Pain Clinic, Qilu Hospital, Cheeloo College of Medicine, Shandong University, Ji’nan, China; ^2^Department of Respiratory, Qilu Hospital, Cheeloo College of Medicine, Shandong University, Ji’nan, China

**Keywords:** cough variant asthma, latent myofascial trigger points, autonomic phenomena, airway hyper-responsiveness, inflammation

## Abstract

**Background:**

Cough variant asthma (CVA) is a chronic inflammatory airway disease characterized by airway hyper-responsiveness (AHR), of which cough is the only symptom. The cough is a result of the contraction of the vocal cords, diaphragm, sternocleidomastoid muscle, and other respiratory related muscles caused by the AHR. Long-term chronic coughing can lead to repetitive contraction and chronic strain of the muscles involved in the head and neck, ultimately contributing to the formation of latent myofascial trigger points (MTrPs). In turn, latent MTrPs can also irritate or compress the nerves around them, triggering cough. The date indicated that latent MTrPs can induce autonomic phenomena and are effective in allergic rhinitis. But their roles in asthma are unclear. In this article, the efficacy and safety of latent MTrPs injection therapy in CVA were investigated.

**Methods:**

This randomized controlled trial was conducted with 110 patients. Patients were assigned to the intervention or control group in a 1:1.5 ratio. Intervention group (*n* = 44): single injection therapy with latent MTrPs. Control group (*n* = 66): budesonide-formoterol plus montelukast for 8 weeks. During the 36-week follow up period, the recurrence rate at week 36, cough visual analog scale (VAS), ACT (asthma control test)-scores, ACQ5 (asthma control questionnaire)-scores, AQLQ (asthma quality of life questionnaire)-scores, proportion of using rescue medication, and adverse events were evaluated.

**Results:**

The recurrence rate at week 36 was lower in the intervention group than in the control group (36 weeks, 5.0 vs. 34.55%, *p* = 0.001). There were significant differences between groups in change from baseline to 36 weeks in VAS [36 weeks, 1.70 (1.49) vs. 3.18 (2.04), *p* < 0.001]; ACT-score [36 weeks, 21.38 (2.65) vs. 18.53 (3.00), *p* < 0.001]; ACQ5-score [36 weeks, 0.85 (0.55) vs. 1.52 (0.62), *p* < 0.001]; AQLQ-score [36w, 174.40 (18.22) vs. 151.69 (24.04), *p* < 0.001]; proportion of using rescue medication (36 weeks, 5.0 vs. 29.1%, *p* = 0.003). Fewer adverse events occurred in the two groups.

**Conclusion:**

Latent myofascial trigger points injection therapy provided long-acting, practical, short treatment duration and safety methods for CVA.

**Clinical Trials Registration:**

http://www.chictr.org.cn/index.aspx, Chinese Clinical Trial Registry Center, ChiCTR2100044079.

## 1. Introduction

Cough variant asthma (CVA) is an atypical form of asthma with cough as the only symptom. It is a common chronic inflammation disease of the airway worldwide, affecting 1–18% of the population in different countries (1). A multicenter prospective observational study shows that approximately one-third of chronic cough is caused by CVA (2, 3). Nevertheless, the mechanisms responsible for the hypersensitivity of the cough reflex are not fully understood. Guidelines recommend that adults or children with CVA regularly use a low dose of inhaled glucocorticoids as maintenance therapy to reduce airway inflammation and AHR (4–6). Pharmacogenomics has shown that genetic polymorphisms can alert the response to bronchodilators in patients with asthma (7). Approximately, 5% of patients with CVA remain poorly controlled despite inhaled glucocorticoids or leukotriene receptor antagonists (8).

Myofascial trigger points (MTrPs) are defined as hyperirritable spots, usually within a taut band of skeletal muscle, that are painful on palpation and can induce referred pain, motor dysfunction, and autonomic phenomena (9). Whether accompanied by spontaneous pain, MTrPs can be divided into active and latent MTrPs (10, 11). In general, active MTrPs cause clinical pain syndromes. Latent MTrPs, on the other hand, can cause non-pain-related symptoms such as depression, anxiety, insomnia, and tinnitus. In addition, in the head and cervical can cause cough, tears, rhinorrhea, salivation, and other autonomic phenomena (12). In a previous study, injection of latent MTrPs has been shown to be effective for the treatment of allergic rhinitis (13).

The pathophysiological mechanisms of CVA remain unclear, however, chronic inflammation and AHR are thought to play an important role. Our previous experience with CVA patients offered two main reasons to explain the possible interaction between CVA and latent MTrPs: (1) palpation of latent MTrPs transiently triggered cough in some patients and (2) significant improvement in cough symptoms within minutes after latent MTrPs injection. Therefore, we suspected that latent MTrPs in the head and neck regions were somewhat correlated to the pathogenesis of CVA. We proposed that latent MTrPs affect the balance of the autonomic nervous system, leading to abnormal contraction of airway smooth muscle. To investigate the efficacy and safety of injection therapy of latent MTrPs in CVA, we conducted an open-label randomized controlled trial.

## 2. Materials and methods

### 2.1. Trial design

The study was an open-label, randomized observer-masked controlled trial to compare the effect of latent MTrPs injection therapy vs. budesonide-formoterol plus montelukast therapy in adult CVA patients. Signed informed consent was obtained from all participants. The study took place at Qilu Hospital, Cheeloo College of Medicine of Shandong University, Jinan, China. This study was approved by the Human Research Ethics Committee of Qilu hospital (KYLL-202011-127) and registered in the Clinical Trial Registry Center (ChiCTR2100044079).

The research included a 2-week placebo run-in phase, a treatment process, and an observational period. A total of 110 patients were randomly assigned utilizing computer access in a 1:1.5 ratio to receive a single injection therapy of latent MTrPs, sternocleidomastoid trigger points, medial or lateral pterygoid muscle trigger points, splenius capitis muscle trigger points, and sternoclavicular joint trigger points (intervention group, *n* = 44) or budesonide-formoterol plus montelukast for 8 weeks (control group, *n* = 66). If patients have long-term poor asthma control during the study, they may use rescue asthma medication. The rescue medication was salbutamol sulfate (GLAXO WELLCOME, *S.A.*: 100 μg/treatment *via* a metered-dose inhaler, maximum usage 400 μg/day as needed). Participants were recruited from the CVA Specialized Outpatient Department of Qilu Hospital, Shandong University. A 36-week of follow up was conducted after treatment.

### 2.2. Patients

Patients who were 18 years of age or older and had a clinical diagnosis of CVA [Global Initiative for Asthma (GINA) 2018 criteria (14, 15)] for at least 8 weeks were included. The diagnosis of CVA was made by a respiratory physician based on history, symptoms, signs, and pulmonary function test. The other inclusion criteria for patients were as follows: (1) <75 years old. (2) Any gender or ethnicity. (3) All patients signed an informed consent form. The exclusion criteria were as follows: (1) Cough caused by pneumonia, upper respiratory tract infections, chronic obstructive pulmonary disease, interstitial fibrosis, or other extra-pulmonary diseases. (2) Patients with known hypersensitivity to lidocaine, vitamin B_12_, or betamethasone. (3) Comorbidity, includes chronic pulmonary, cardiovascular, renal, neurologic, or other systemic disease. (4) Long-term use of oral glucocorticoids within the last 3 weeks. (5) Smoking within the past 6 months. (6) Pregnancy.

### 2.3. Procedures

#### 2.3.1. Intervention group

##### 2.3.1.1. Latent MTrPs injection procedures

The drugs used for latent MTrPs injection containing vitamin B_12_ (JinYao Corp, Tianjin City, China; 2 ml:1 mg), 2% lidocaine injection (ZhaoHui Corp, Shanghai City, China; 5 ml:100 mg), and compound betamethasone injection (MSD Merck Sharp & Dohme AG, Switzerland; 1 ml: 5 mg betamethasone dipropionate and 2 mg betamethasone sodium phosphate) were diluted to 20 ml with 0.9% saline for a single injection. Injection of latent MTrPs was performed using needle 25 (0.5 mm × 36 mm) and a 20 ml syringe (We Go Corp, Weihai City, China).

**Figure 1:**
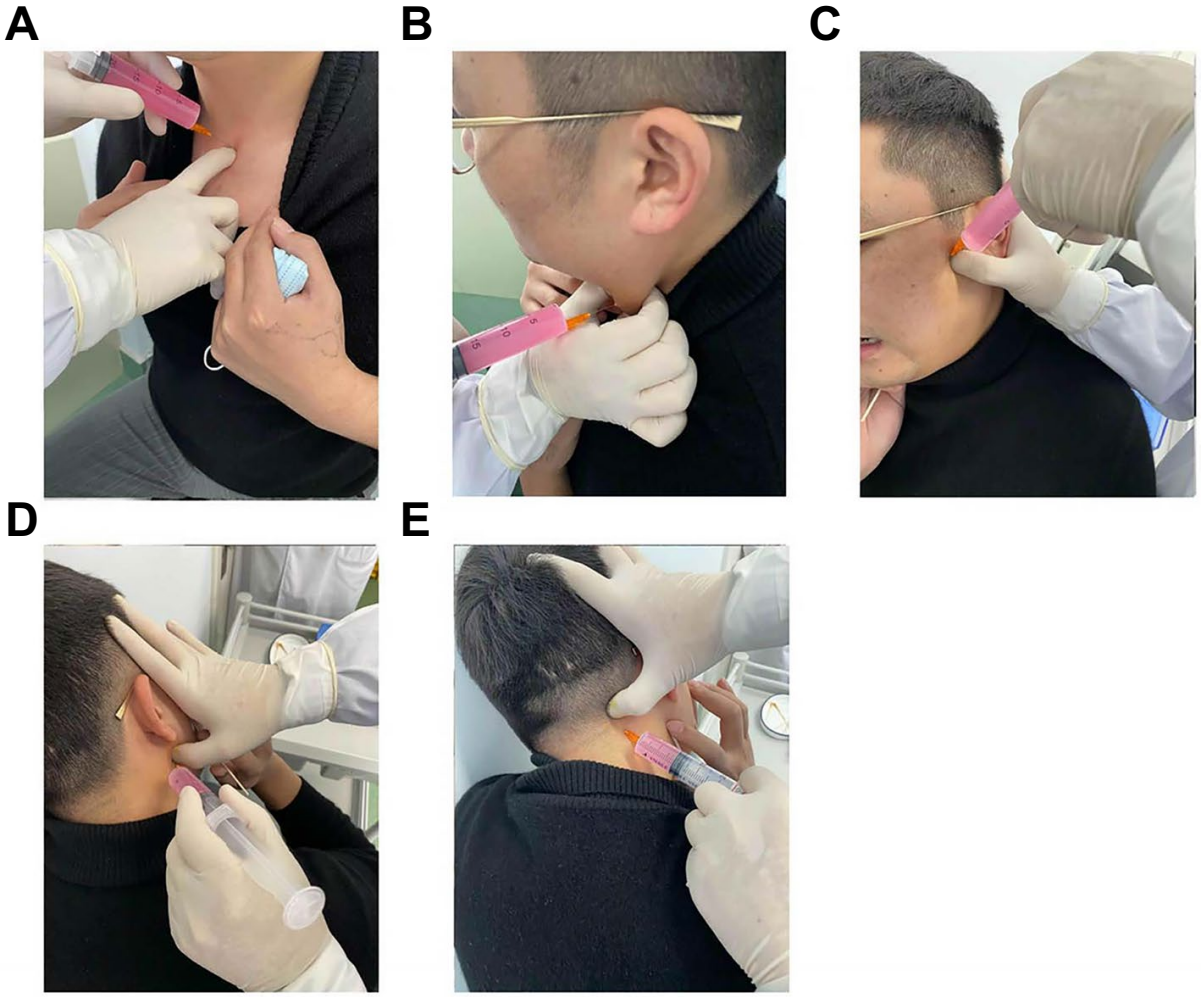
Latent MTrPs injection for CVA. Sternoclavicular joint latent MTrPs injection **(A)**. Sternocleidomastoid muscles latent MTrPs injection **(B)**. Medial or lateral pterygoid muscles latent MTrPs injection **(C,D)**. Splenius capitis muscles latent MTrPs injection **(E)**. MTrPs, myofascial trigger points; CVA, cough variant asthma.

The latent MTrPs were mainly found in the sternoclavicular joint, sternocleidomastoid, medial or lateral pterygoid muscles, and splenius capitis muscles by palpation (Figure 1). However, it was difficult to palpate when some trigger points were hidden in muscles, and the final therapeutic effects depend on the accuracy of palpated points (16). Accurate signs of latent MTrPs can be confirmed by the patient showing “jumping signs,” which may include head retraction, fascial (or forehead) wrinkles, verbal responses, or local twitch responses (LTRs) (11, 12). Palpation and injection of latent MTrPs were performed according to Travell and Simons’ “Trigger Point Manual” (17).

##### 2.3.1.2. Latent MTrPs in the medial or lateral pterygoid muscles

Palpation was performed with progressive and continuous deep pressure with a finger on the skin surface to identify latent MTrPs in the medial or lateral pterygoid muscles. Patients were instructed to remain seated and to immobilized the head and shoulders to maximize relaxation of the muscle being palpated (18). Once the trigger points were identified, the thumb of one hand of the therapist remained in a fixed position on the skin, disinfected the surrounding skin, had the patients keep their mouths open (to prevent the needle from blocking in the temporomandibular joint) and with the other hand inserted the syringe into the muscle, adjusted the depth of the needle, and when the patients felt a referred pain indicating that the needle had been inserted into the latent MTrPs, 3–5 mL of a liquid medication was then injected. There were many small blood vessels near the muscles. To avoid intravascular injection, it was necessary to withdraw before injection. The other side was injected using the same technique.

##### 2.3.1.3. Latent MTrPs in the sternocleidomastoid muscles

For palpation of the sternocleidomastoid muscle, the patient was placed in a sitting position with the head slightly tilted to one side, and the examiner pinched the muscle with the thumb and index finger to identify the latent MTrPs. Using the same technique, both sides of the sternocleidomastoid muscles were injected.

##### 2.3.1.4. Latent MTrPs in the splenius capitis muscles

We had the patients sit on the table with both forearms crossed and place the forehead resting on the forearms to identify the trigger point by palpation. The injection technique was the same as above, with pressure on the injection sites after injection to promote better absorption of the drug.

##### 2.3.1.5. Latent MTrPs in the sternoclavicular joint

When palpating the sternoclavicular joint, we had the patient sit upright and located the trigger point by palpation. Care was taken to avoid accidental injection of medication into the subclavian artery. Palpation and injection were performed by the same professor who has 30 years of experience.

#### 2.3.2. Control group

Budesonide and formoterol fumarate powder for inhalation (AstraZeneca AB: 160 ug budesonide and 4.5 μg of formoterol per inhaler, one inhaler every 12 h) plus montelukast (Merck Sharp & Dohme Ltd.: 10 mg per night) for 8 weeks.

### 2.4. Outcomes

The primary outcome was recurrence rate at week 36. The secondary outcomes mainly included cough VAS, ACQ-5 scores, ACT scores, and AQLQ scores at each of the measurement times points; the proportion of patients using rescue medication at week 36. Scores were obtained before treatment (baseline) and 1, 2, 4, 8, 12, 24, and 36 weeks after treatment.

The VAS is a validated summative score for cough variant asthma, ranging from 0 to 10 points, with VAS with 1–3, 4–6, and 7–10 points indicate mild, moderate, and severe cough, respectively (19). Recurrence was defined as the patient’s 36 weeks cough VAS score returning to or being higher than the baseline level. The “Asthma Control Questionnaire” (ACQ) is a very common questionnaire used to assess asthma control. The ACQ-5 includes five questions about asthma symptoms in the past week (20). Each score ranges from 0 (no impairment) to 6 points (maximum impairment). The ACT includes five questions about asthma symptoms in the past 4 weeks, with each score ranging from 1 (maximum impairment) to 5 points (no impairment). The AQLQ contains 29 questions about asthma symptoms and social and psychological problems during the past 1 week. Each item is scored on a scale from 1 (severe impairment) to 7 points (no impairment).

Safety was evaluated according to the type and severity of adverse events (*A.E.s*). Data on *A.E.s* are collected from the start of treatment until the end of follow-up. Patients were asked to record any local or systemic effects during the observation period.

### 2.5. Statistical analysis

Prior to conducting the study, a pre-experiment was performed, by which we obtained a 36 weeks recurrence rate of 11.1% in Group I and 45.5% in Group C. Setting *α* = 0.05, *β* = 0.1, and an allocation ratio of 1:1.5, we calculated at least 24 patients in group I and 36 patients in group C using the PASS software. Assuming a 20% lost rate, we calculated at least 30 patients in group I and 45 patients in group C. Date were analyzed using independent samples *t*-tests, paired-samples *t*-test, Chi-square test or Fisher’s exact test. For statistical analysis, we used SPSS version 22.0 software and GraphPad Prism 8.3, and measured data are described as mean (SD). A *p* value less than 0.05 was considered to indicate statistical significance.

## 3. Results

### 3.1. Patients

**Figure 2:**
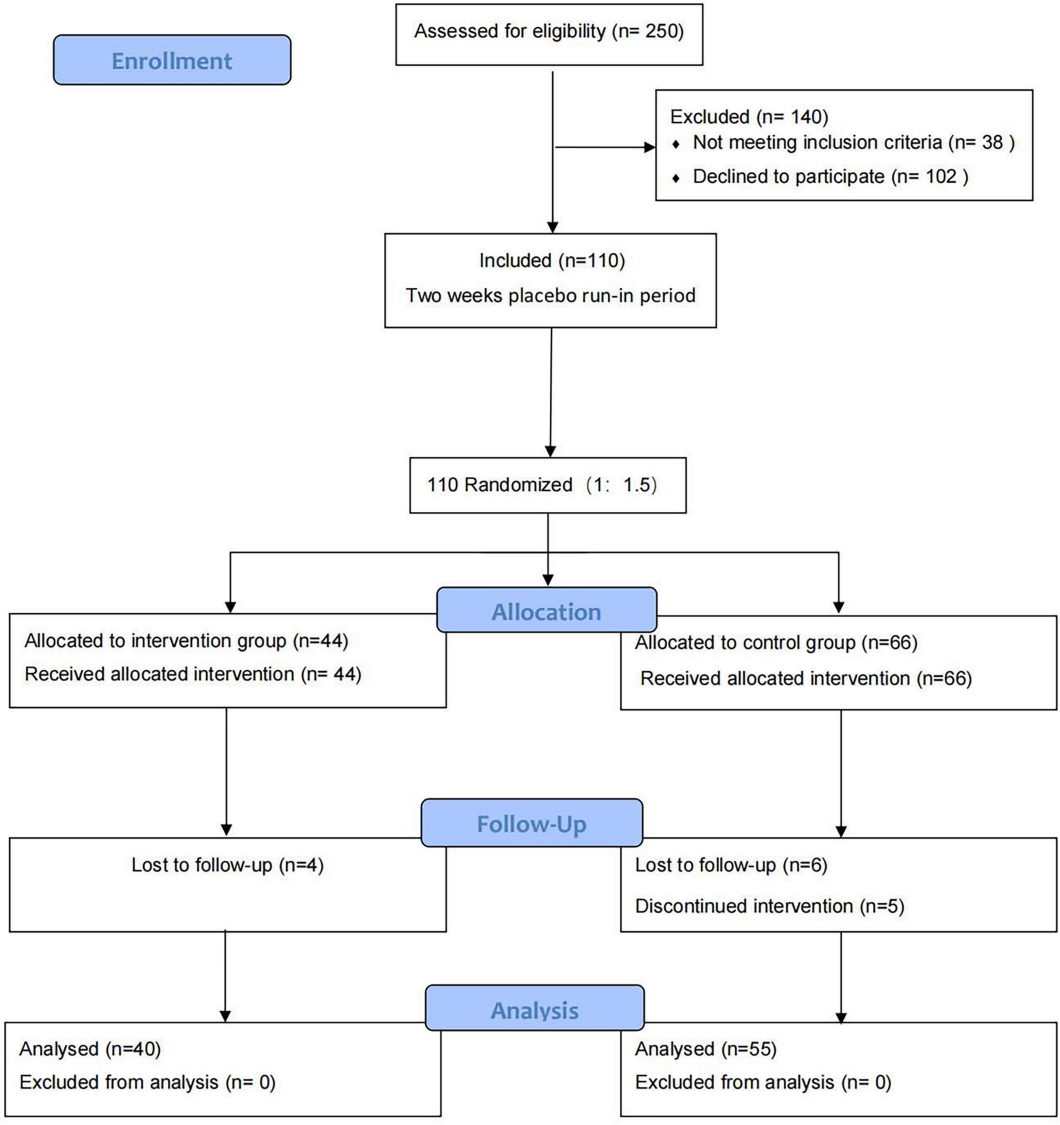
Flow diagram of trial procedure.

According to the recruitment strategy, a total of 110 patients who had CVA were enrolled in the study. Of them, 95 (86.4%) participants completed the trial and were included in the full analysis. Ten patients were lost to follow up, including 4 (9%) in the intervention group and 6 (9%) in the control group. Five patients failed to persist on budesonide-formoterol. The flow diagram of the participant is shown in Figure 2. The two groups were similar with respect to baseline characteristics (*p* > 0.05; Table 1).

**Table 1:** Demographic characteristics of participants.

Characteristics	Intervention group (*n* = 40)	Control group (*n* = 55)	*p* value
Sex, *n* (%)
Male	15 (37.5)	22 (40.0)	0.805
Female	25 (62.5)	33 (60.0)	0.805
Age, mean (SD)	45.56 (12.04)	41.62 (14.06)	0.154
Education attainment, *n* (%)
Primary school and lower	3 (7.5)	8 (14.5)	0.563
Middle and high school	23 (57.5)	30 (54.5)	0.563
College and higher	14 (35.0)	17 (30.9)	0.563
Cigarette smoking, *n* (%)
No smoking	31 (77.5)	40 (72.1)	0.845
Ever smoking	7 (17.5)	11 (7.3)	0.845
Passive smoking	2 (5.0)	4 (20.0)	0.845
Family history of CVA, *n* (%)	4 (10)	2 (3.6)	0.208
VAS score, mean (SD)	5.80 (1.20)	5.52 (1.21)	0.281
ACQ-5 score, mean (SD)	2.35 (0.68)	2.37 (0.50)	0.855
ACT score, mean (SD)	12.78 (1.86)	13.09 (1.97)	0.432
AQLQ score, mean (SD)	119.65 (17.55)	126.22 (15.58)	0.058

Values are presented as *n*, mean (SD), or percent. CVA, cough variant asthma; ACT, asthma control test; ACQ-5, asthma control questionnaire-5; VAS, visual analog scale; AQLQ, asthma quality of life questionnaire; and SD, standard deviation.

### 3.2. Primary outcomes

Overall, recurrence occurred in 21 of 95 participants (22.11%) at week 36, 2 of 40 (5%) in the intervention group vs. 19 of 55 (34.55%) in the control group [36 weeks, −0.296 (95%CI, −0.4388 to −0.1533), *p* = 0.001; Table 2].

**Table 2:** Recurrence rate and proportion of rescue medication used at week 36.

Group, No. (%)
Outcomes	Intervention group (*n* = 40)	Control group (*n* = 55)	Rate difference (95% CI)	*p* value
Primary outcome
Recurrence at 36 weeks	2 (5.00)	19 (34.55)	−0.296 (−0.4388 to −0.1533)	0.001
Secondary outcomes
Adverse events	3 (7.50)	8 (14.55)	−0.0705 (−0.1944 to 0.0534)	0.462
Proportion of using rescue medication at 36 weeks	2 (5.00)	16 (29.10)	−0.241 (−0.3787 to −0.1033)	0.003

Values are presented as *n*, percent. *p* value represents the Chi-square test comparing changes between the intervention group and the control group. CI, confidence interval.

### 3.3. Secondary outcomes

**Figure 3:**
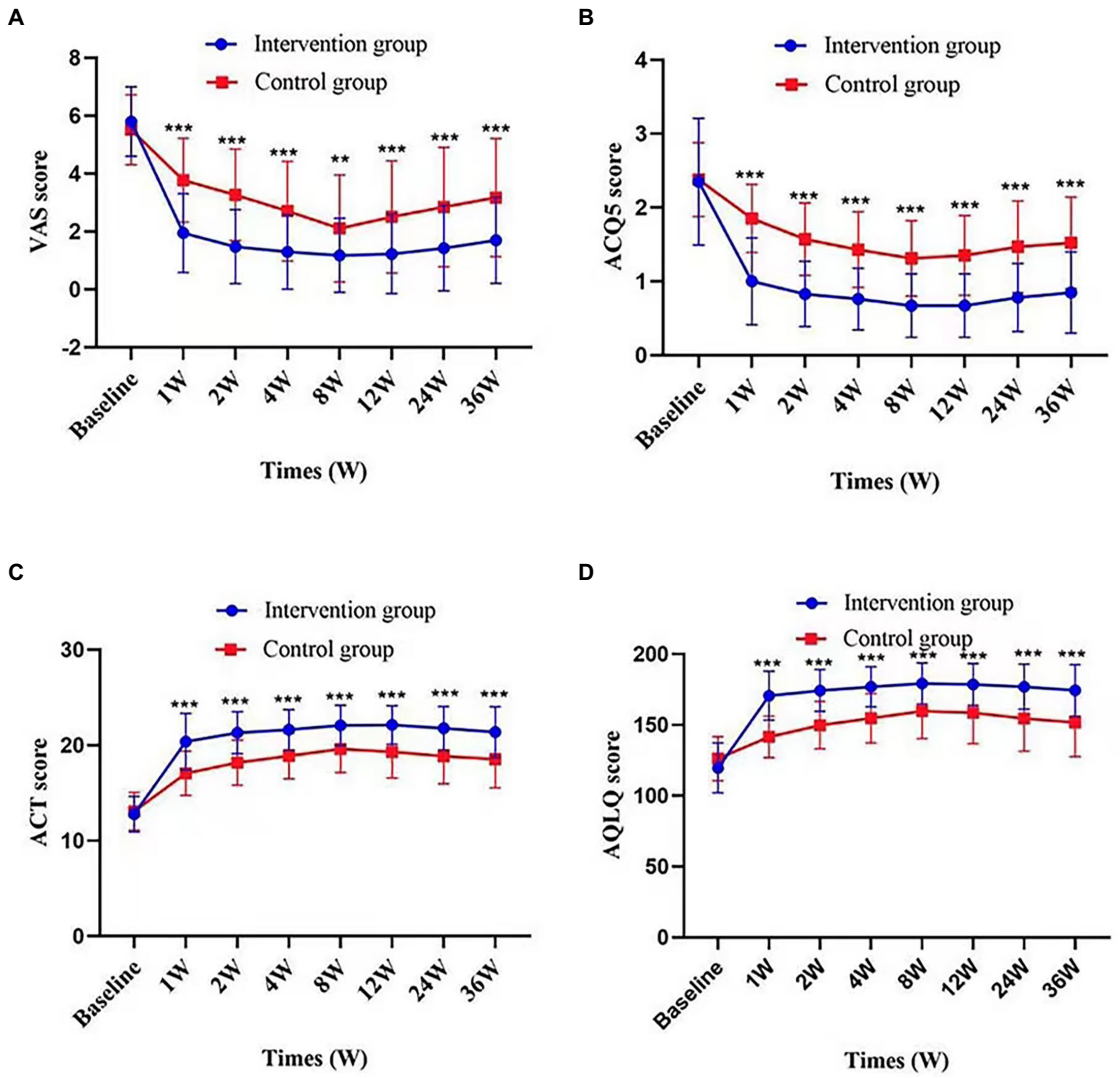
Change in VAS score, ACQ5score, ACT score, and AQLQ score according to times and groups. Figures demonstrate values of VAS score **(A)**, ACQ5 score **(B)**, ACT score **(C)**, and AQLQ score **(D)** at each time point in intervention group and control group. Values are presented as mean (SD). *p* value represents the independent samples *t*-tests comparing changes between the two groups. ^**^p value <0.01; ^***^p value <0.001, comparison between the two groups.

**Figure 4:**
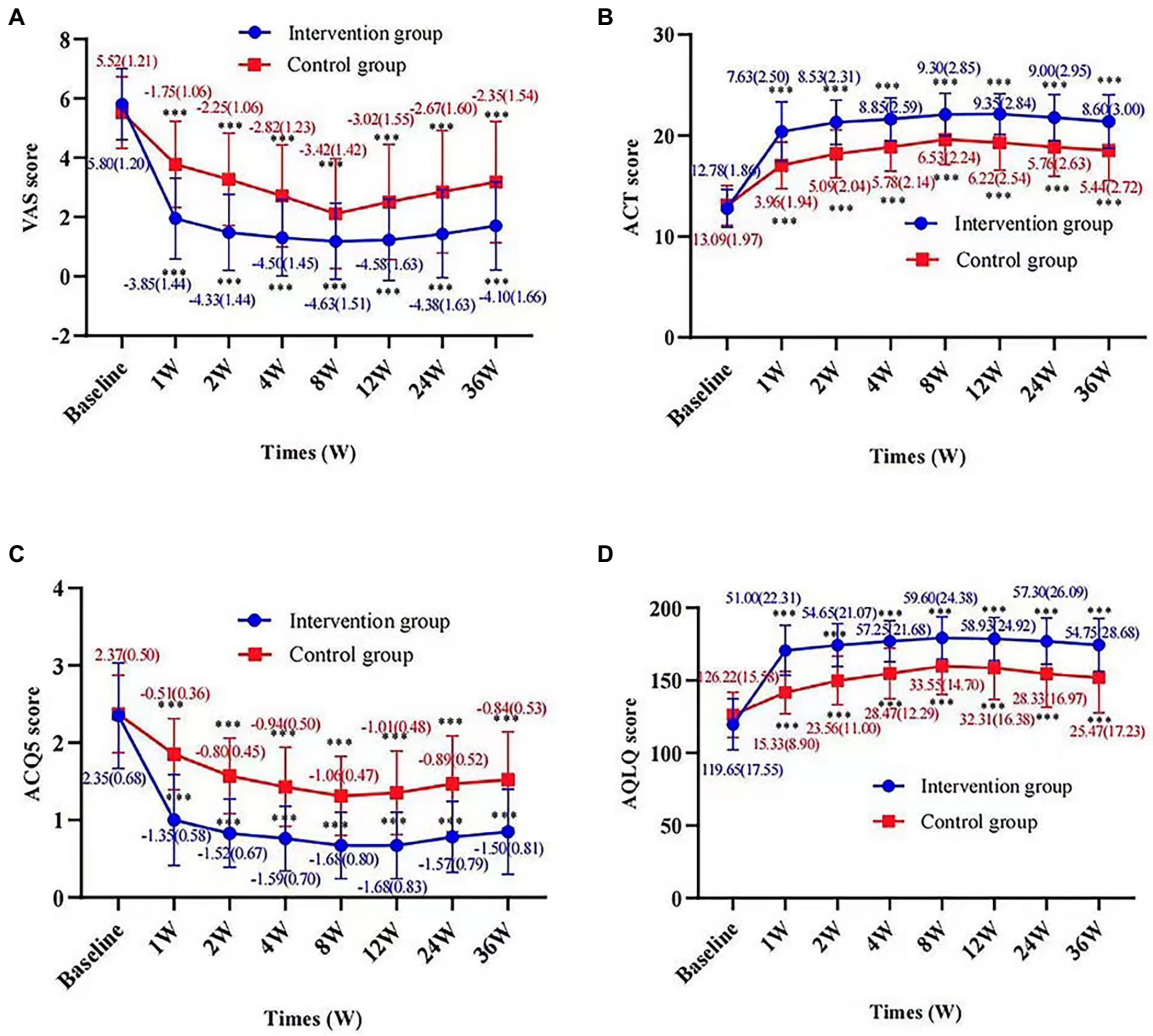
Change in VAS score, ACT score, ACQ5 score, and AQLQ score according to times and groups. Figures show the difference in VAS score **(A)**, ACT score **(B)**, ACQ5 score **(C)**, and AQLQ score **(D)** at each time point from baseline for the intervention and control groups, respectively. Values are presented as mean (SD). *p* value represents a paired sample *t*-test for the change from baseline in the two groups. ^***^p value <0.001, compared with baseline.

After treatment, the intervention group showed a significant VAS reduction compared with the control group throughout the follow-up period. The improvement in cough symptoms was observed in the first week of treatment and persisted until the end point. The results of efficacy analysis are shown in Table 3 and Figure 3A. From week 8 to the end of the follow-up period, cough VAS tended to increase in both groups, but at week 36, there was a significant improvement in cough symptoms in both the intervention and control groups compared with baseline [−4.10 (1.66) and − 2.35 (1.54), *p* < 0.001, Figure 4].

**Table 3:** The differences between groups in change from baseline to 36 weeks in outcomes.

	Intervention group (*n* = 40)	Control group (*n* = 55)	Mean difference, 95%CI	*p* value
Baseline
VAS score	5.80 (1.20)	5.52 (1.21)	0.28 (−0.226 to 0.772)	0.281
ACQ5 score	2.35 (0.68)	2.37 (0.50)	−0.02 (−0.263 to 0.219)	0.855
ACT score	12.78 (1.86)	13.09 (1.97)	−0.31 (−1.111 to 0.479)	0.432
AQLQ score	119.65 (17.55)	126.22 (15.58)	−6.57 (−13.351 to 0.214)	0.058
Week 1
VAS score	1.95 (1.36)	3.78 (1.45)	−1.83 (−2.414 to −1.249)	<0.001
ACQ5 score	1.00 (0.59)	1.85 (0.46)	−0.85 (−1.067 to −0.642)	<0.001
ACT score	20.40 (2.95)	17.05 (2.32)	3.35 (2.271 to 4.420)	<0.001
AQLQ score	170.65 (17.21)	141.55 (14.60)	29.10 (22.606 to 35.603)	<0.001
Week 2
VAS score	1.48 (1.28)	3.27 (1.58)	−1.79 (−2.395 to −1.200)	<0.001
ACQ5 score	0.83 (0.44)	1.57 (0.49)	−0.74 (−0.935 to −0.547)	<0.001
ACT score	21.30 (2.20)	18.18 (2.36)	3.12 (2.170 to 4.066)	<0.001
AQLQ score	174.30 (14.80)	149.78 (16.74)	25.52 (17.935 to 31.101)	<0.001
Week 4
VAS score	1.30 (1.29)	2.71 (1.72)	−1.41 (−2.021 to −0.797)	<0.001
ACQ5 score	0.76 (0.42)	1.43 (0.51)	−0.67 (−0.863 to −0.481)	<0.001
ACT score	21.63 (2.11)	18.88 (2.39)	2.75 (1.831 to 3.673)	<0.001
AQLQ score	176.90 (14.14)	154.69 (17.40)	22.21 (15.774 to 28.645)	<0.001
Week 8
VAS score	1.18 (1.28)	2.11 (1.85)	−0.93 (−1.572 to −0.296)	0.005
ACQ5 score	0.67 (0.43)	1.31 (0.51)	−0.64 (−0.832 to −0.450)	<0.001
ACT score	22.08 (2.10)	19.62 (2.49)	2.46 (1.518 to 3.396)	<0.001
AQLQ score	179.25 (14.50)	159.76 (19.51)	19.49 (12.558 to 26.414)	<0.001
Week 12
VAS score	1.23 (1.37)	2.51 (1.94)	−1.28 (−1.959 to −0.610)	<0.001
ACQ5 score	0.67 (0.43)	1.35 (0.54)	−0.68 (−0.880 to −0.485)	<0.001
ACT score	22.13 (2.02)	19.31 (2.75)	2.82 (1.844 to 3.788)	<0.001
AQLQ score	178.58 (14.75)	158.53 (21.71)	20.05 (12.614 to 27.481)	<0.001
Week 24
VAS score	1.43 (1.48)	2.85 (2.06)	−1.42 (−2.151 to −0.708)	0.001
ACQ5 score	0.78 (0.46)	1.47 (0.62)	−0.69 (−0.913 to −0.472)	<0.001
ACT score	21.78 (2.28)	18.85 (2.90)	2.93 (1.863 to 3.977)	<0.001
AQLQ score	176.95 (15.92)	154.55 (23.07)	22.40 (14.459 to 30.350)	<0.001
Week 36
VAS score	1.70 (1.49)	3.18 (2.04)	−1.48 (−2.200 to −0.763)	<0.001
ACQ5 score	0.85 (0.55)	1.52 (0.62)	−0.67 (−0.922 to −0.435)	<0.001
ACT score	21.38 (2.65)	18.53 (3.00)	2.85 (1.669 to 4.027)	<0.001
AQLQ score	174.40 (18.22)	151.69 (24.04)	22.71 (14.097 to 31.322)	<0.001

Values are presented as mean (SD). *p* value represents the independent samples *t*-tests comparing changes between the intervention group and the control group.

SD, standard deviation; CI, confidence interval; VAS, visual analog scale; ACQ5, asthma control questionnaire-5; ACT, asthma control test; and AQLQ, asthma quality of life questionnaire.

There were also significant differences between groups in change from baseline to 36 weeks in ACQ-5 score, ACT score, AQLQ score (*p* < 0.001); proportion using rescue medication [36 weeks, −0.241 (95%CI, −0.3787 to −0.1033), *p* = 0.003]. Changes in these outcomes on the prespecified end points are summarized in Tables 2, 3 and Figure 3. Adverse events were rare and generally not attributed to intervention therapy (Tables 2, 4). A total of 11 *A.E.s* were classified as mild. Intervention group: disturbances of glucose level (2, 5%); headache (1, 2.5%). Control group: insomnia (3, 5.5%); drowsiness (2, 3.6%); anxiety (1, 1.8%); rash (1, 1.8%); palpitation (1, 1.8%). Two patients developed a transient hyperglycemia after latent MTrPs injection, with blood glucose levels were 13.6 and 14.1 mmol/L, respectively, and this phenomenon was disappeared the day after the injection therapy.

**Table 4:** Adverse events related to treatment.

Adverse events, *n* (%)	Intervention group (*n* = 40)	Control group (*n* = 55)
Anxiety	0 (0)	1 (1.8)
Rash	0 (0)	1 (1.8)
Insomnia	0 (0)	3 (5.5)
Headache	1 (2.5)	0 (0)
Drowsiness	0 (0)	2 (3.6)
Disturbances of glucose level	2 (5)	0 (0)
Palpitation	0 (0)	1 (1.8)

Values are presented as *n*, per cent.

## 4. Discussion

This study was the first trial to evaluate the efficacy and safety of latent MTrPs injection in CVA. Latent MTrPs injection therapy showed significant improvement in asthma symptoms at all time points, especially at week 8 and week 12. Although budesonide-formoterol plus montelukast therapy is effective to some extent in CVA, cough VAS increased from week 8 and the increase was statistically significant at weeks 12, 24, and 36 of follow-up compared with week 8. This study also suggests that latent MTrPs injection therapy may address patient’s overdependence on or poor adherence to maintenance therapy with inhaled of glucocorticoids. There are no serious adverse events attributable to latent MTrPs injection therapy or budesonide-formoterol plus montelukast therapy; two patients reported a transient hyperglycemia after latent MTrPs injection treatment, but they were alleviated quickly. On the one hand, this phenomenon may be related to betamethasone. On the other hand, it might be associated with the nervous stress of the patient during the needling to make the sympathetic nerves relatively excited (21).

### 4.1. Relationship between CVA and latent MTrPs

The pathogenesis and pathological process of CVA have not been fully elucidated. Previous studies have shown that transient receptor potential vanilloid 1 (TRPV1) and endogenous neuropeptides are closely associated with the development of CVA (22–26). According to Simons’ theory, latent MTrPs reside in asymptomatic areas and trigger referenced pain and autonomic phenomena only upon stimulation (17). Previous research has established that many inflammatory cytokines are expressed in the vicinity of MTrPs (27), including bradykinin (BK), capsaicin, substances P (SP), α-tumor necrosis factor, serotonin, histamine, norepinephrine, and extracellular fluid. The fluid contains hydrogen ions that can induce the release of calcitonin gene-related peptides (CGRP) *via* motor terminals and nociceptive muscle receptors (28). Some endogenous substances such as SP, CGRP, BK can not only activate TRPV1 directly, but also phosphorylate the specific structure of TRPV1, lowing its threshold and sensitizing TRPV1 (29, 30). The depolarizing current generated by the activation of TRPV1 is conducted to the cough center of the nucleus solitaire *via* the vagus nerve (31). The relief of CVA symptoms after injection of latent MTrPs gave us two main hypotheses for a possible interaction between the physiopathology of CVA and latent MTrPs: (1) the rebalancing between sympathetic and parasympathetic nerves and (2) the subsiding of neurogenic inflammations at MTrPs.

We also found that injection of latent MTrPs into the head and neck muscles was more effective than trigger points in the extremities or shoulders in relieving cough symptoms. This may further suggest that neuroinflammation and imbalances in the autonomic nerve network of the head and neck region, especially sympathetic and parasympathetic nerves, are the main mechanisms causing airway smooth muscle contraction and AHR.

### 4.2. Relief CVA symptoms through latent MTrPs injection

Various neuropeptides released by neurogenic inflammation have different effects on CVA (32). The CGRP can dilate the blood vessels by acting on CGRP receptors in airway blood vessels, causing congestion and edema of the airway mucosa (33). SP acts on the NK1 receptor, increasing airway microvascular permeability and promoting infiltration of plasma proteins and inflammatory cells. In addition, SP stimulates airway smooth muscle and induces AHR. Latent MTrPs injection therapy alleviated the neurogenic inflammations at the MTrP, decreased the production of endogenous peptides such as SP and CGRP, and balanced the autonomic nerve network, especially the cervical sympathetic and vagus nerves, thereby restoring the airway mucosal function.

We also examined whether the improvements in symptoms were related to the drug used for the injection. Lidocaine has been reported to have anti-inflammatory properties (34). Its potential mechanisms may include blocking vagus reflex and inhibiting smooth muscle contraction. Betamethasone is a long-acting corticosteroid with anti-inflammatory and anti-allergic effects, including reduce inflammatory exudation and improve local blood flow. The longest duration of any drugs we used did not last more than 4 weeks. But, in our observations, we have found that the relief of symptoms can persist for tens of weeks. Consequently, we attribute the relief of cough to the alleviation of neurogenic inflammation at the MTrP by the injection of latent MTrPs rather than to the effect of the inject drugs themselves.

### 4.3. Relationship between latent MTrPs injection and traditional acupuncture

Research has shown that acupuncture can improve lung ventilation and anti-inflammatory activity and regulate the neuroendocrine network (35), however, we consider them to be different. First, acupuncture points are located at specific positions on the meridian. In contrast, latent MTrPs are hyperirritable points, usually within a taut band of skeletal muscle; the location of the point is individual and may vary from person to person. Second, acupuncture points in traditional Chinese medicine have pathological and physiological properties, while trigger points have only pathological properties. Moreover, trigger points injection focus on inducing LTRs.

Although the study has obtained certain research results, there are some limitations of this research. Firstly, the outcome variable may be subject to unmasked administration of therapy. In terms of the implementation of blinding methods, the injection of latent MTrPs is special, and it is difficult to achieve double blindness, it was only a randomized, observer-masked controlled trial. Secondly, outcome indicators are relatively subjective and susceptible to other factors. Thirdly, the long-term side-effects of latent MTrPs injection were not evaluated in this study. In future studies, we would add objective indexes such as FeNO or lung function tests.

## 5. Conclusion

In the pilot study, we suggested that latent MTrPs injection therapy provided long-acting, practical, short treatment duration and safety methods for CVA. We inferred that the inflammatory of latent MTrPs might play an essential role in the pathogenesis of CVA. The potential mechanism may be that neurogenic inflammations at MTrPs disrupt the balance between sympathetic and parasympathetic nerves in the autonomic nervous system, result in airway smooth muscle contraction and airway hyper-responsiveness.

## Data availability statement

The original contributions presented in the study are included in the article/supplementary material, further inquiries can be directed to the corresponding author.

## Ethics statement

This study was approved by the Human Research Ethics Committee of Qilu hospital. The patients/participants provided their written informed consent to participate in this study. Written informed consent was obtained from the individual(s) for the publication of any potentially identifiable images or data included in this article.

## Author contributions

QL: conceptualization, methodology, validation, resources, writing–original draft, and writing–review and editing. WZ: conceptualization, methodology, investigation, data curation, and writing–original draft. TT: validation, formal analysis, and data curation. YL and HB: conceptualization and methodology. QH: formal analysis, data curation, writing–original draft, and visualization. FQ: conceptualization, methodology, validation, resources, writing-original draft, writing–review and editing, supervision, project administration, and funding acquisition. All authors contributed to the article and approved the submitted version.

## Funding

This study was funded in part by the National Natural Science Foundation of China (No. 81672250) and the Fundamental Research Funds of Shandong University.

## Conflict of interest

The authors declare that the research was conducted in the absence of any commercial or financial relationships that could be construed as a potential conflict of interest.

## Publisher’s note

All claims expressed in this article are solely those of the authors and do not necessarily represent those of their affiliated organizations, or those of the publisher, the editors and the reviewers. Any product that may be evaluated in this article, or claim that may be made by its manufacturer, is not guaranteed or endorsed by the publisher.

## Abbreviations

CVA: Cough variant asthma
MTrPs: Myofascial trigger points
LTRs: Local twitch responses
AHR: Airway hyperresponsiveness
VAS: Visual analog scale
ACT: Asthma control test
ACQ5: Asthma control questionnaire-5
AQLQ: Asthma quality of life questionnaire
*A.E.s*: Adverse events
TRPV1: Transient receptor potential vanilloid 1
SP: Substance P
CGRP: Calcitonin gene-related peptide
BK: Bradykinin
NK1: Neurokinin 1
